# *H. pylori* clinical isolates have diverse *babAB* genotype distributions over different topographic sites of stomach with correlation to clinical disease outcomes

**DOI:** 10.1186/1471-2180-12-89

**Published:** 2012-05-30

**Authors:** Shew-Meei Sheu, Bor-Shyang Sheu, Wen-Cheng Chiang, Cheng-Yen Kao, Hsiu-Mei Wu, Hsiao-Bai Yang, Jiunn-Jong Wu

**Affiliations:** 1 Institute of Basic Medical Sciences, College of Medicine, National Cheng-Kung University, Tainan, Taiwan; 2 Institute of Molecular Medicine, College of Medicine, National Cheng-Kung University, Tainan, Taiwan; 3 Department of Internal Medicine, College of Medicine, National Cheng Kung University, 138 Sheng Li Road, Tainan, Taiwan; 4 Department of Medical Laboratory Science and Biotechnology, College of Medicine, National Cheng-Kung University, Tainan, Taiwan; 5 Department of Pathology, Ton-Yen General Hospital, Hsinchu, Taiwan; 6 Department of Internal Medicine, National Cheng Kung University Hospital, College of Medicine, National Cheng Kung University, 138 Sheng Li Road, Tainan, Taiwan

## Abstract

**Background:**

Intragenomic recombination between *babA* and *babB* mediates antigenic variations and may help *H. pylori* colonization. This study determined whether variable genotypes of *babA* and *babB* correlate to different clinical disease outcomes, and can distribute over the different gastric niches.

**Results:**

This study enrolled 92 clinical strains (45 from peptic ulcer, 27 from gastritis, and 20 from gastric cancer) to detect whether the *babA* and *babB* are at locus A or B by PCR reactions using the primers designed from the upstream and variable region of the *babA* and *babB* genes. Four genotypes of *babA* and *babB* (A B, AB B, A AB, AB AB) were found. The distribution of the 4 genotypes in 92 clinical strains was significantly different among patients with different gastric diseases (*p* < 0.05). The isolates from gastric cancer patients had a higher rate of AB AB genotype than those from non-cancer patients (40.0% *vs.* 9.7%, *p* < 0.05). The AB AB genotype was associated with a higher intensity of intestinal metaplasia (*p* < 0.05), but did not correlate with a higher inflammation and colonization density in gastric histology (*p* > 0.05). Besides, the study enrolled 19 patients to verify whether variable genotypes of *babAB* existed in the different gastric niches. Among the patients infected with more than one *babAB* genotypes over antrum and corpus, there were higher rate of genotypes as A B or AB AB in isolates from antrum than in those from corpus (75.0 % *vs.* 16.7%, *p* < 0.05).

**Conclusions:**

The *H. pylori* isolate with the AB AB genotype correlates with an increased gastric cancer risk, and colonize in an antrum predominant manner.

## Background

*Helicobacter pylori* infection increases the risk of peptic ulcers and gastric adenocarcinoma of the human stomach [1-3]. *H. pylori* adherence to the gastric epithelium and deliver effectors to induce inflammation [4,5]. One of the best-studied adhesins is the blood group antigen binding adhesin (BabA), which binds Lewis b (Leb) and related ABO antigens [6,7]. Putative adhesin, BabB, is encoded by *babB*, which shares nearly identical N- and C-terminal sequences with *babA*[7,8]. The reversed chromosomal locations of *babA* and *babB* between strain J99 and 26695 prove the recombination events between these two genes [9,10]. The two genes also show both geographic and allelic variation [11]. Moreover, the duplication of *babA* or *babB* gene is mediated by gene conversion between the different chromosomal loci [12-14]. Bäckström *et al*. [14] demonstrated that the silent *babA* gene of a Leb-nonbinding strain can be activated by recombination into the *babB* gene. The rhesus monkey model showed that most recovered isolates have replaced *babA* with a second copy of *babB* after several weeks of infection [12].

In western countries, patients infected with *babA*-positive *H. pylori* isolates are associated with an increased risk of peptic ulcer diseases [15,16]. However, this association is not confirmed in patients from the Eastern Asia, or some other western countries [17-19]. Colbeck *et al*. [20] used PCR to detect whether the downstream of *hpyD* (locus A) and *s18* (locus B) are *babA* or *babB* and found single-colony isolate with mixed *babA* and *babB* genotype at the same locus, indicating subpopulations within the bacterial population derived from a single colony. It is worthy to answer whether the genetic profiles of *babA* and *babB* could be related to the different clinical disease outcomes or the specific *H. pylori*-related histological features.

There are different predominant cell types in the antrum and corpus. The parietal cells producing HCl locate in the corpus and make a different pH gradient to the antrum. Our previous study showed patients with chronic *H. pylori* infection expressed a higher intensity of Lewis b in the gastric epithelium of corpus than in the antrum [17]. Recombination between *babA* and *babB* might help *H. pylori* to change its adhesion ability to adapt different niches within the stomach [21]. Accordingly, it is worthy to determine the genotype distribution of *babA* and *babB* in the *H. pylori* infection over the different topographic locations as either antrum or corpus in human stomach. In this study, we analyzed the clinical significance of *babA* and *babB* genotypes and the presence of *babA* and *babB* at locus A and B of multiple colonies from different gastric niches to understand the *babAB* genetic profile of *H. pylori* isolates across gastric regions within the same host.

## Results

### Distributions of ***babA*** and ***babB*** genotypes in patients with different clinical diseases

**Figure 1:**
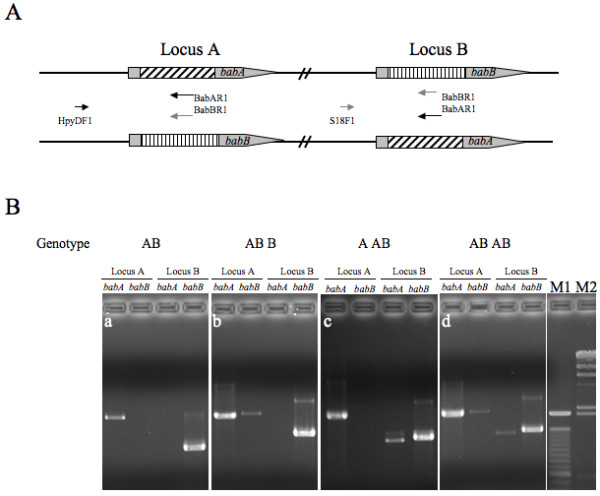
**PCR banding patterns of *babAB* genotypes. (A)** Primer pairs used for gene detection at locus A and B. The forward primers, HypDF1 and S18F1, located in the upstream region of *babA* or *babB*, are paired with BabAR1 or BabBR1 primers to determine whether the gene at locus A and B is *babA* or *babB*. **(B)** PCR banding patterns of genotype A B, AB B, A AB and AB AB. The AB B genotype showed two bacterial populations in the single-colony isolate, the dominant as *babA* and the minor as *babB*, at locus A. The strain with A AB genotype represented a dominant population of babB and a minor population of *babA* at locus B. The combination of AB B and A AB was defined as an AB AB genotype. Lane M1, a 100 bp molecular marker; lane M2, l HindIII marker. The size of PCR products at locus A and B was 2.1-2.6 kb and 1.0-1.5 kb, respectively.

Detection of *babAB* genotypes was based on the primer design shown in Figure 1. Among 92 strains, the distribution of the four genotypes (A B, AB B, A AB and AB AB) was 46 (50%), 21 (22.8%), 10 (10.9%), and 15 (16.3%), respectively. There was no difference in the gender distribution among the different genotypes (Chi-square test, *p* > 0.05). The mean age of patients infected with genotype as AB AB was marginally older than those infected with other genotypes (57.6 vs. 50.3 years, Independent-sample t test, *p* = 0.09). The distributions of the four genotypes were significantly different in the patients with different clinical diseases (Table 1, Chi-square test, *p* = 0.04). The mean age of GC patients was higher than the other non-cancer patients (58.6 *vs.* 49.5 years, Independent-sample t test, *p* = 0.01). The rate of the AB AB genotype in the patients with GC was higher than that in the three groups of non-cancer patients (40.0% [8/20] *vs.* 9.7% [7/72], Fisher exact test, *p* < 0.05, odds ratio: 6.2; 95%CI: 1.9-20.3).

**Table 1:** **The*babA*and*babB*genotypes of*H. pylori*from different clinical patient groups**

**N (%)**	**DU** **(n = 18)**	**GU** **(n = 27)**	**Gastritis** **(n = 27)**	**GC** **(n = 20)**	**Total** **(n = 92)**	**Statistics** ***p* value**
AB	11 (61.1)	14 (51.9)	15 (55.6)	6 (30)	46 (50)	0.039
AB B	6 (33.3)	4 (14.8)	7 (25.9)	4 (20)	21 (22.8)	
A AB	0 (0)	4 (14.8)	4 (14.8)	2 (10)	10 (10.9)	
AB AB	1 (5.6)	5 (18.5)	1 (3.7)	8 (40)	15 (16.3)	

DU: duodenal ulcer. GU: gastric ulcer. GC: gastric cancer. The difference between the four genotypes in gastric diseases was assessed by the Chi-square test.

As the AB AB genotype was higher in the patients with gastric cancer, we further tested whether such a genotype may lead to a higher rate of precancerous changes or more severe histological inflammation. In the patients with GC, the AB AB genotype was associated with a higher intensity of intestinal metaplasia (IM) in the antrum compared to non-AB AB genotype (2.0 *vs.* 0.27, *p* = 0.02). However, in the non-cancer patients, the AB AB genotype wasn’t associated with higher acute inflammation scores (sum of antrum, corpus and cardia: 3.43 *vs.* 2.94, *p* > 0.05), chronic inflammation scores (sum of antrum, corpus and cardia: 7.29 *vs.* 7.22, *p* > 0.05), *H. pylori* density (sum of antrum, corpus and cardia: 8.14 *vs.* 8.84, *p* > 0.05), or the intensity of IM (0.43 *vs.* 0.51, *p* > 0.05) compared to non-AB AB genotype.

### Difference in the ***babA*** and ***babB*** genotype between isolates from antrum and corpus

For the 19 patients who provided isolates from different gastric niches over the antrum and corpus, the genotype composition of *babA* and *babB* at locus A and B of their antrum and corpus isolates is shown in Table 2. Four patients (no. 7, 12, 29, 30) were infected with an A B genotype strain across the antrum and corpus, and 15 patients were found to have a mixed genotype strains (AB B, A AB or AB AB) in only the corpus, or both gastric niches. In those 15 patients, 3 patients (no. 28, 2, 4) had the same mixed genotypes across the antrum and corpus. Eight of remaining 12 patients had one major genotype across both gastric niches. Combining with the 7 patients (no. 7, 12, 29, 30, 28, 2, 4) with only one genotype, 78.9% (15/19) of our patients had one major genotype distributed across two niches.

**Table 2:** **The genotype compositions of antrum and corpus*H. pylori*isolates from 19 patients**

**Case No.**	***babAB* genotype of isolates**		**Major *babAB* genotype across antrum & corpus**	**Dominant genotype in the antrum / corpus**	**A B or AB AB as dominant genotype of 12 patients infected with more than one genotype**	
	**antrum (n)**	**corpus (n)**			**antrum**	**corpus**
**7**	A B (4)	A B (4)	A B	A B / A B	x	x
**12**	A B (4)	A B (4)	A B	A B / A B	x	x
**29**	A B (4)	A B (4)	A B	A B / A B	x	x
**30**	A B (4)	A B (4)	A B	A B / A B	x	x
**28**	A AB (4)	A AB (4)	A AB	A AB / A AB	x	x
**2**	AB AB (4)	AB AB (4)	AB AB	AB AB / AB AB	x	x
**4**	AB AB (4)	AB AB (4)	AB AB	AB AB / AB AB	x	x
**1**	AB AB (3)	AB AB (2)	AB AB	AB AB / -	+	-
	AB B (1)	AB B (2)				
**3**	AB B (3)	AB B (4)	AB B	AB B / AB B	-	-
	A B (1)					
**6**	AB AB (3) A AB (1)	AB AB (4)	AB AB	AB AB / AB AB	+	+
**10**	AB AB (3)	AB B (4)	-	AB AB / AB B	+	-
	AB B (1)					
**19**	A B (3)	A B (3)	A B	A B / -	+	-
		A AB (4)				
**21**	A B (4)	A B (3)	A B	A B / A B	+	+
		AB B (1)				
**25**	A B (4)	A B (1)	-	A B / AB B	+	-
		AB B (3)				
**14**	A B (4)	A B (2)	A B	A B / -	+	-
	AB B (2)	AB B (2) AB AB (2)				
**24**	A B (3)	A B (1)	-	A B / -	+	-
	A AB (1)	AB B (1)				
		A AB (2)				
**27**	A AB (3)	A B (1)	A AB	A AB / A AB	-	-
	AB AB (1)	A AB (3)				
**11**	A B (4)	A AB (2)	-	A B / -	+	-
	AB B (1)	AB AB (3)				
	AB AB (2)					
**17**	A B (3)	A B (3)	A B	- / -	-	-
	AB B (3)	A AB (1)				
	A AB (2)	AB AB (2)				

Patient no. 1, 3, 6, 19 and 21, who were infected by 2 genotypes, still have a major one across both gastric niches, and that was also true in 2 (no. 14, 27) patients having 3, and 1 (no. 17) patient having 4 genotypes represented in their infections. - indicating that the patients have non-dominant *babA* and *babB* genotype in the isolates of antrum or corpus. The patients’ number was according to our previous study [22].

Among those 12 patients infected with more than one genotype (Table 2), the frequency of the major dominant genotype, A B combined with AB AB, in the antrum was higher compared with that in the corpus (75% [9/12] *vs.* 16.2% [2/12], *p* = 0.012, odds ratio: 15). However, 6 of 12 patients lacked a dominant genotype in their corpus isolates.

### Sequence analysis and comparison

**Figure 2:**
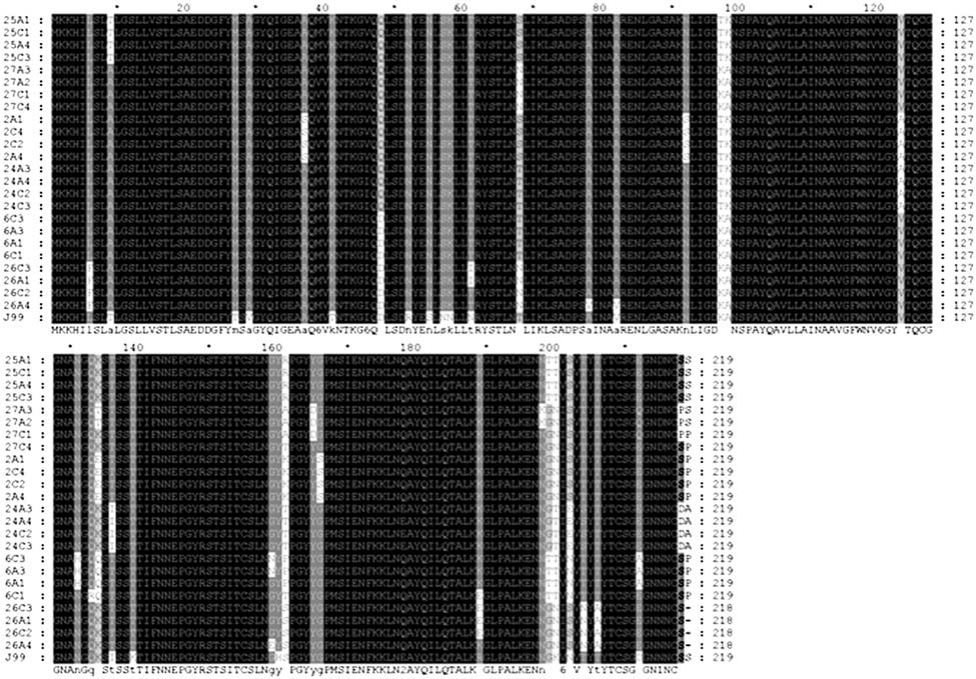
**The *babA* sequences at locus A of the antrum and corpus isolates.** Cardinal numbers indicate different patients’ isolates. A1-4 and C1-4 were single-colony isolate isolated from the antrum and the corpus, respectively. White highlighting indicates amino acids different from consensus.

At locus A, each patient’s antrum and corpus isolates had specific substitutions of amino acids in the region of BabA (Figure 2 and Table 3). However, there was no obvious difference between the antrum and corpus isolates in the sequencing region, except from patient no. 27 (amino acid 134 and 198). We also found 5 different nonsynonymous substitutions at amino acid 161 in 6 patients’ isolates, as compared with strain J99. The same scenario (sequence specificity in individual patients’ strains but not between the antrum and corpus isolates) was in the *babB* sequences.

**Table 3:** **The amino acid substitutions in BabA encoded by*babA*at locus A**

	**The location of amino acid substitutions**															
**Case No.**	6	9	37	92	131	134	136	161	165	166	198	202	204	206	212	218
**25**	L	**T**	A	N	N	K	T	**R**	Y	G	N	S	T	T	E	S
**27**	L	A	A	N	N	T, K	T	**A**	**D**	G	N, K	S	T	T	**Q**	**P**
**2**	L	A	**S**	**S**	N	**E**	T	**K**	Y	**S**	N	S	T	T	E	S
**24**	L	A	A	N	N	K	**I**	**T**	Y	G	N	**E**	**S**	T	E	**D**
**6**	L	A	A	N	**H**	**Q**	T	**P**	Y	G	N	**N**	T	T	**A**	S
**26**	**F**	A	A	N	N	K	T	**S**	Y	G	N	S	**N**	**A**	E	S

### CT repeats of ***babA*** and ***babB*** at locus B

Genes at locus B are regulated by CT repeats in the 5’ coding region, and the number of CT repeats (5, 8 and 11) make in-frame protein expression possible [12,20]. The CT repeats of the *babB* gene at locus B are shown in Table 4. The corpus isolates in 7 of 12 patients had a change of CT repeats of the *babB* gene at locus B, and antrum isolates of those patients always have the same CT repeats, except patient 17 (Table 4).

**Table 4:** **The number of CT repeats in the 5’ coding region of*babB*at locus B**

**Case No.**	**Antrum isolates (n = 2)** **(CT repeat number)**	**Corpus isolates (n = 2)** **(CT repeat number)**	**Concordance**	**Change of CT repeat in the corpus**
**2**	8, 8	8, 8	+	-
**12**	8, 8	8, 8	+	-
**24**	7, 7	7, 7	+	-
**30**	11, 11	11, 11	+	-
**1**	8, 8	7, 10	-	+
**11**	8, 8	7, 9	-	+
**26**	8, 8	8, 9	-	+
**6**	9, 9	9, 12	-	+
**21**	7, 7	9, 10	-	+
**27**	9, 9	9, 8	-	+
**14**	8, 8	7, 7	-	-
**17**	7, 10	8, 7	-	+

Four patients (no. 2, 12, 24, 30) were infected by isolates with one kind of CT repeat (7, 8 and 11) across the antrum and corpus, but only one of them (no. 24) had an out of frame *babB*. In the other patients (no. 1, 11 and 26), their antrum isolates contained 8 CT repeats but the corpus isolates changed to 7, 9 or 10 repeats. For the patients (no. 6, 21 and 27) who were infected by the antrum isolates with 7 or 9 CT repeats, their corpus isolates also had a change of CT repeats, but the number of CT repeats was still out of frame (9, 10 and 12), except in one isolate from patient no. 27 (CT repeats = 8).

### Genotype and BabA expression

**Figure 3:**
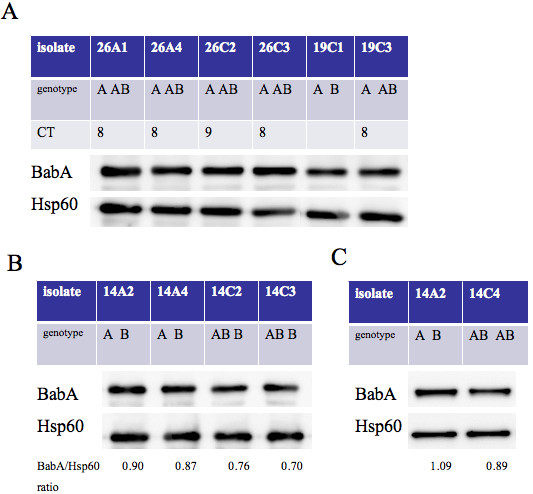
***babA*at locus A dominantly determined BabA expression. (A)** Effect of *babA* at locus B on the *BabA* expression.The isolate (19C3) had *babA* at locus A and in-frame CT repeats of *babA* at locus B, which were compared with the isolate only having *babA* at locus A (19C1). The presence of *babA* at locus A and B was in the isolates 26A1, A4, C2 and C3, but C2 had an out of frame *babA* at locus B. **(B)** Effect of mixed genotype at locus A on the *BabA* expression. The isolates from one patient (no. 14) had a mixed genotype at locus A (14C2 and C3), which was compared with those with *babA* only at locus A (14A2 and A4). **(C)** Comparison of *BabA* between AB AB and A B genotypes. Hsp60 was as an internal control.

To determine the effect of *babA* at locus A and B on BabA expression (Figure 3A), we found that the *babA* at locus B didn’t obviously affect the level of BabA expression, when compared to the isolates 19C3 (A AB) and 19C1 (A B). All the isolates (26A1, A4, C2 and C3) had the A AB genotype, but the CT repeats of the *babA* at locus B of C2 was out of frame. The expression of BabA was not affected by whether *babA* at locus B was in or out of frame. We further determined whether a mixed genotype at locus A would affect BabA expression, and found 14C2 and 14C3 with the AB B genotype (BabA/Hsp60 ratio: 0.76 and 0.70) had slightly lower expression than 14A2 and 14A4 with the A B genotype (BabA/Hsp60 ratio: 0.90 and 0.87, Figure 3B). AB AB genotype also had the lower BabA expression than A B (BabA/Hsp60 ratio: 1.09 and 0.89, Figure 3C).

## Discussion

The occurrence of intragenomic recombination between *babA* and *babB* has been demonstrated in *in vitro* and *in vivo* experiments, implicating this mechanism may possibly assist *H. pylori* to adapt in the human stomach [12,14]. In addition, mixed genotypes of *babA* and *babB* at locus A or B have been demonstrated [20]. The clinical association of the *babA* and *babB* genotype of *H. pylori* strains and genetic profile with infections of the antrum and corpus of a single host are still unclear. In this study, we demonstrated that the AB AB genotype, one dominant genotype in the antrum, was associated with the precancerous lesion as IM, and correlated with gastric cancer. However, *H. pylori* infection by such AB AB genotype has not lead into a more dense colonization or inflammation severity in gastric histology. Our data indicate *H. pylori babA* and *babB* genotypes as AB AB should at least exert with better adaptation to gastric environment during carcinogenesis.

Colbeck *et al*. [20] found 9 genotypes (A B, AB B, A AB, A A, B B, B A, B C, C B and B AB) in their study. Nevertheless, our study only found four genotypes (A B, A AB, AB B and AB AB) in the 168 isolates from 19 patients’ antrum and corpus (Table 2). It indicates the genotype diversity of *babAB* in Taiwanese isolates could be obviously less complicate. Moreover, at least one *babA* gene at locus A existed in each of the isolates. This finding is compatible with our previous report to reveal the Taiwanese *H. pylori* isolates are nearly 100% *babA*-positive [17], and support the higher prevalence of *babA* in *H. pylori* strains from East Asian countries than those from western worlds [23]. Moreover, Matteo *et al*. [24] demonstrated that 9 of 34 patients (26.5%) had *bab* gene variation across the antrum and corpus of a single host at a specific time point. We found that 12 of 19 patients (63.2%) infected by more than one genotype in either one or both gastric niches. The prevalence discrepancy between two studies could be due to the analysis of *bab* genotype from the bacterial pool or single-colony isolate.

Analysis of the sequences of *babA* and *babB* revealed that nonsynonymous substitutions of amino acids occurred between the individual strains (Figure 2, Table 3 and data not shown), but did not differ between the gastric niches. Pride *et al*. [11] also showed high allelic diversity within *babA* and *babB* in the strains from different patients. Judging by the 6 different nonsynonymous substitutions of amino acid 161 in the 6 patients’ strains, that codon was a highly variable site. This is worth further investigation, as it may be a special site responsible for adapting to differences in individual stomachs.

CT repeats in the 5’ coding region of *babA* and *babB* are more commonly found at locus B than locus A [20]. We found that the corpus isolates had a higher frequency of changes in number of CT repeats of *babB* at locus B than the antrum isolates (Table 4). Among those 7 patients infected by the corpus isolates with a change of CT repeats, only one (patient no. 27) had the isolate changing CT repeats to in-frame (CT = 8) (Table 4). This data indicates that BabB expression could be tightly controlled by phase variation due to out of frame repeats in the corpus. Among 12 patients infected by isolates with more than one genotype, their isolates from antrum have a higher rate of A B and AB AB as dominant genotypes than corpus (9/12 *vs.* 2/12, *p* < 0.05). Moreover, half of those patients lacked a dominant genotype in their corpus isolates. These results suggest the environment in the corpus may favor different adaptation for the isolates with different *H. pylori* genetic diversities.

The presence of the AB AB genotype was higher in GC patients with older age (Table 1). In addition, the AB AB genotype is not correlated with more severe inflammation or precancerous changes in the non-cancer patients. Based on this cross-sectional clinical histological data, it suggests the AB AB strains may have a better adaptation to the cancerous environment in stomach, instead of leading into more toxicity in gastric carcinogenesis. In Figure 3A, we show that the *babA* gene at locus A dominantly determines BabA expression, and the mixed genotype as AB at locus A may decrease the BabA expression (Figure 3B and 3C). It is thus possible a mixed genotype as AB at locus A may make *H. pylori* isolates to contain a subpopulation losing BabA expression. Alternatively, the mixed genotype as AB at locus B may possibly allow *H. pylori* to change BabB expression and thus deserves further study.

In addition, our previous data have shown that the intensity of Lewis b become decreased in antrum atrophy, but can be preserved in corpus to mediate higher colonization of bug overthere [17]. So, it shall be also implicative to test whether the AB AB genotype dominantly in antrum can have advantage to adapt the gastric epithelium with weak Lewis b expression in future.

## Conclusions

The *H. pylori* isolate with *babA* and *babB* genotype as AB AB genotype correlates with an increased gastric cancer risk, and colonize in an antrum predominant manner. Such AB AB genotype shall be associated with a better adaptation to the gastric precancerous or cancer environment, and possibly generate subpopulations losing BabA or BabB.

## Methods

### Patients and bacterial isolates

A total of 92 *H. pylori* strains were cultured from the biopsy specimens of patients with different clinical diseases as duodenal ulcer (DU, n = 18), gastric ulcer (GU, n = 27), gastritis (n = 27), or gastric cancer (GCA, n = 20), defined by endoscopy with histological confirmation. All patients had given informed consent and underwent panendoscopy in our institute. During panendoscopy for each patient, five bits of gastric biopsy, including 2 from the antrum, 2 from the corpus, and 1 from the cardia were obtained. The bacterial culture and histological examination were applied as the previous article [17]. This study was approved by 'Human Experiment and Ethics Committee of National Cheng Kung University Hospital' (No. HR-98-023).

Single-colony isolates from the antrum and corpus were randomly picked from the primary culture plates. For each site, at least 3-4 single-colony isolates were randomly selected to determine the *babAB* genotype. Colony culture for DNA extraction was less than 8 passages to decrease the possibility of genetic variation in vitro. Each of the 19 patients infected in the antrum and corpus by isolates with the same RAPD banding pattern was described previously [22].

### Detection of ***babA*** and ***babB*** genotypes

The detection of *babA* and *babB* genotypes was based on the method of Colbeck *et al*[20]. HypDF1-BabAR1 and HypDF1-BabBR1 primers were used to determine whether the gene at locus A was *babA* or *babB*. In the same way, S18F1-BabAR1 and S18F1-BabBR1 primers were applied to determine whether the gene at locus B was *babA* or *babB* (Figure 1A). The 40 cycles of amplification reactions were performed with 20 pmoles of primer, 0.15 mM each deoxynucleoside triphosphate, reaction buffer with MgCl_2_ and 1 U Taq DNA polymerase (New England Biolabs, Beverly, MA, USA) in a final volume of 50 μl. The conditions of thermal cycling were described previously [20]. Each amplified product (20 μl) was analyzed on a 1% agarose gel stained with ethidium bromide.

### Genotype definition

The *babA* and *babB* genotype of each single-colony isolate was based on the previous description [20]. A J99-like isolate showed the expected PCR bands of *babA* at locus A and *babB* at locus B and was defined as the “A B genotype” (Figure 1B-a). A single-colony isolate containing both *babA* and *babB* at the same locus was defined as “mixed genotype” (such as AB B, A AB, and AB AB), indicating that there were subpopulations within the bacterial population derived from a single colony. An isolate with an AB B genotype contained one population with *babA* and the other population with *babB* at the same locus A (Figure 1B-b). The A AB genotype represented two bacterial populations, the dominant one with *babB* and the minor one with *babA* at locus B, although both derived from a single colony (Figure 1B-c). A mixed genotype detected at both locus A and B was defined as an AB AB (Figure 1B-d). A minor band from *babB* at locus B could be non-specific binding because its size is larger than the prediction.

### Sequencing

The PCR products were sequenced by using either the BabAR1 or BabBR1 primer, depending on the amplification of *babA* or *babB*. The sequencing was conducted by the Mission Biotech Company, Taipei, Taiwan.

### Western blot

*H. pylori* grew for 2 days, was harvested, and suspended in ddH_2_O. We used the Bio-Rad Protein Assay (Bio-Rad Laboratories, Hercules, CA, USA) to determine and adjust protein concentration of each bacterial suspension. An equal amount (2μg) of bacterial protein was loaded to perform SDS-PAGE and a 1:2000 dilution of anti-BabA polyclonal antibody (Ab, a gift from Prof. Odenbreit) was used in a western blot [17]. The detection of BabA protein was performed with Super Signal® West Pio Chemiluminescent substrate (Thermo Fisher Scientific Inc., Rockford, IL, USA) and exposed in an LAS-3000 imaging system (Fujifilm, Tokyo, Japan).

### Statistics

Statistical analysis was performed by the Chi-square test, Fisher exact test, Mann-Whitney U test and Student’s t test as appropriate. The difference was considered significant with a *p* value less than 0.05.

## Authors' contributions

Dr. Sheu MS has made contributions to the experimental design, acquisition and analysis of data. Dr. Sheu BS and Dr. Wu JJ coordinated the conduct of the whole study and made interpretation of data. Chiang WC, Kao CY and Wu HM conducted the acquisition of data. Dr. Yang HB reviewed the pathology. All authors read and approved the final manuscript.
